# Evidence Against Effects of Cultural Group and Prior Knowledge on Feature Binding in Working Memory

**DOI:** 10.5334/joc.390

**Published:** 2024-07-23

**Authors:** Hiu Wah Cheung, Nicolas Geeraert, Vanessa M. Loaiza

**Affiliations:** 1 Department of Psychology, University of Essex, School of Computer Science and Electronic Engineering, University of Essex, United Kingdom; 2 Department of Psychology, University of Essex, United Kingdom; 3 School of Psychology, University of Sheffield, United Kingdom

**Keywords:** working memory, cultural differences, binding, unitisation, prior knowledge

## Abstract

Feature binding is the process of integrating features, such as colour and shape, into object representations. A persistent question in the literature concerning whether feature binding is an automatic or resource-demanding process may depend on unitisation, that is, whether the to-be-bound information is intrinsic (belonging to) or extrinsic (contextual). Given extensive evidence showing that Easterners may process information more holistically than Westerners, such cultural differences may be useful to understand the fundamental processes of feature binding in visual working memory (WM). Accordingly, we recruited British and Chinese participants to complete a visual WM task wherein to-be-remembered colours were integrated within (i.e., intrinsic binding) or as backgrounds (i.e., extrinsic binding) of to-be-remembered shapes (Experiments 1 and 2). Experiment 2 further investigated the role of prior knowledge in long-term memory to facilitate feature binding in WM. During retrieval, participants decided among three probes: a target, a lure (i.e., recombination of the presented features), and a new colour/shape. Hierarchical Bayesian multinomial processing tree models were fit to the data to estimate parameters representing binding and item memory. The current results suggest that intrinsic and extrinsic binding memory are similar between the two cultural groups, with no prior knowledge benefits for either intrinsic or extrinsic binding for either cultural group. This result conflicts with the Analytic and Holistic framework and suggests that there are no cultural differences or prior knowledge benefits in feature binding.

Working memory (WM) is the system that keeps a limited amount of information temporarily active (e.g., a few coloured shapes; Cowan, 1999; Luck & Vogel, 1997). A persistent question in the visual WM literature concerns whether feature binding, i.e., integrating different features into an object representation, is an automatic or resource-demanding process. Some findings indicate that memorising more features per object (Luck & Vogel, 1997) or imposing a distracting task (Allen et al., 2006) does not disproportionately impair feature binding, thus suggesting that it is relatively automatic. Conversely, other studies have shown conjunction costs, such that memory is poorer for combinations (e.g., remembering a red apple) compared to individual features (e.g., remembering red and apple individually; Johns & Mewhort, 2002; Parra et al., 2009; Treisman et al., 1977). This signals that feature binding may require cognitive resources.

Whether feature binding is automatic may depend on unitisation, that is, whether the to-be-bound information is intrinsic (i.e., part of the stimulus itself, e.g., its colour) or extrinsic (i.e., not part of the stimulus, e.g., its background; Asch et al., 1960; Ceraso, 1985, 1990; Ecker et al., 2013; Wilton, 1989). Extrinsic binding has been suggested to be more resource-demanding than intrinsic binding given that it requires integrating objects with other contextual, spatial, or temporal elements (Troyer & Craik, 2000). For example, previous studies have shown that intrinsic feature information automatically influences object recognition. Conversely, the reintegration of extrinsic contextual information is more controlled, thus suggesting that extrinsic features are not automatically bound (Ecker, Zimmer, & Groh-Bordin, 2007a, 2007b; Groh-Bordin et al., 2006; Zimmer & Ecker, 2010). The goal of the current work was to investigate whether manipulating cultural differences (Experiments 1 and 2) and prior knowledge in long-term memory (Experiment 2) may inform this long-standing theoretical discussion regarding the nature of feature binding in WM.

## Cultural differences in perception and memory

There is ample evidence that culture affects perception and associated memory processes (e.g., Chua et al., 2005; Nisbett et al., 2001). According to the analytic and holistic framework (Masuda & Nisbett, 2001; Nisbett et al., 2001; Nisbett & Miyamoto, 2005), Westerners (e.g., people from North America and Western Europe) are more analytic in thinking style, such that an object is perceived and identified through its key features (i.e., intrinsic features) and separated from its context (i.e., extrinsic features). In contrast, Easterners (e.g., people from East Asia) are more holistic in thinking style, such that objects are perceived as an integral part of their context. This means that binding extrinsic features is more automatic and akin to intrinsic binding for Easterners compared to Westerners. Consequently, the framework predicts that Westerners focus on and remember foreground objects, whereas Easterners focus on and remember foreground objects and their backgrounds similarly.

Consistent with this prediction, prior work has demonstrated that object recognition memory was relatively unaffected by any background change for Westerners, whereas Easterners were less likely to recognise the target objects when tested with different backgrounds to what had been initially presented during encoding (Chua et al., 2005; Masuda & Nisbett, 2001). These cultural differences have not been considered in the WM literature, but they may inform the previous discussion regarding unitisation and feature binding, such that extrinsic binding is less automatic for Westerners versus Easterners. Moreover, the potential dissociation between extrinsic and intrinsic binding may likewise clarify what drives the previously described cultural differences in perception and memory performance. In other words, these cultural differences may reflect more basic differences in the relative automaticity of extrinsic binding in WM, thus helping to inform the underlying mechanisms of the observed differences between Westerners and Easterners.

## Prior knowledge benefit

There is some evidence showing that prior knowledge from long-term memory boosts WM performance (e.g., Oberauer et al., 2017), particularly for bindings between presented information (e.g., Bartsch & Shepherdson, 2020; Loaiza & Srokova, 2020). Thus, prior knowledge may enhance WM by facilitating the construction of extrinsic bindings specifically, whereas intrinsic bindings may be unaffected, given that they are more automatically established in WM. Furthermore, if extrinsic binding is more automatic in Easterners than in Westerners, then prior knowledge may facilitate extrinsic binding for Westerners. However, if extrinsic and intrinsic bindings are similarly automatic, then prior knowledge should similarly benefit intrinsic and extrinsic binding in both cultural groups.

## Present study

**Figure 1:**
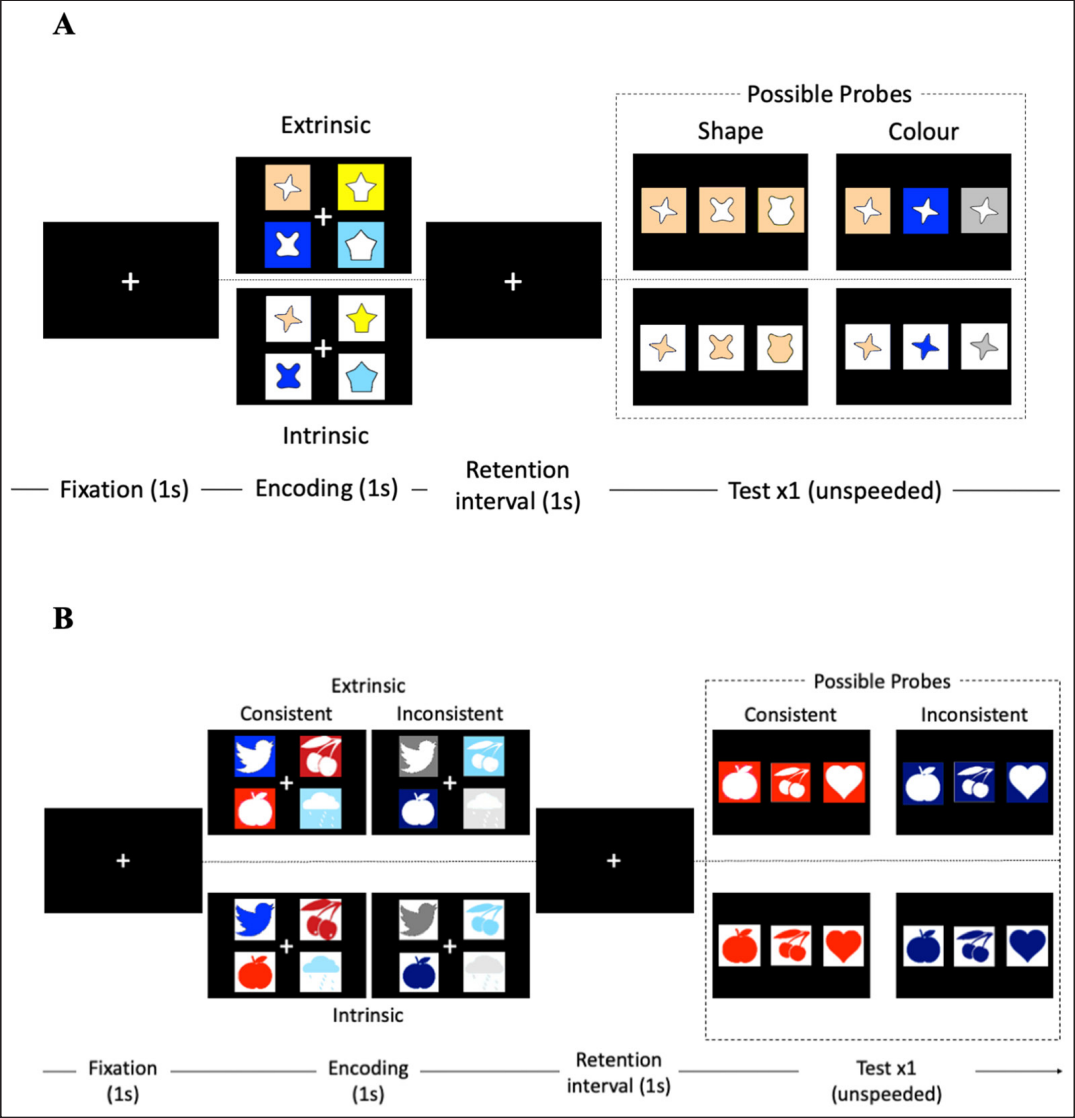
Schematic of the Task in Experiment 1 (Panel A) and 2 (Panel B).

Based on this background, we recruited participants from the UK (Western) and China (Eastern) to take part in two visual WM experiments (see Figure 1 and Table 1).^1^ Experiment 1 concerned whether Easterners show greater extrinsic binding memory than Western participants given previous work suggesting that Easterners process extrinsic information more automatically (Chua et al., 2005; Masuda & Nisbett, 2001). For intrinsic binding memory, one of two possible patterns is likely to emerge. If intrinsic binding is automatic for Easterners, then intrinsic binding memory should be similar between Easterners and Westerners. Alternatively, Easterners’ focus on context may be at the expense of intrinsic binding, in which case Westerners should have greater intrinsic binding memory than their Eastern counterparts. Moreover, much of the prior work has limited analyses to observed task performance, which does not directly reflect item (i.e. individual features) or binding (i.e. the combination of features) memory. Therefore, this study expanded upon prior work by applying hierarchical Bayesian multinomial processing tree (MPT) modelling to derive and analyse latent cognitive parameters of item and binding memory.

**Table 1:** Sample Details and Exclusions.


SAMPLE DETAILS	EXPERIMENT 1		EXPERIMENT 2	
	
	BRITISH	CHINESE	BRITISH	CHINESE

Total N attempted	70	82	49	77

N failed to pass the colour blindness/ demographic screening phase	3	2	5	0

N excluded for pre-registered reasons:	37	50	14	46

1. Technical issues	0	0	0	0

2. Incomplete data (e.g., from quitting early)	11	12	11	17

3. Mismatch of culture or place of birth	26	18	2	6

4. Stayed outside their birth country for more than 3 years	0	20	1	23

Final N for analysis after pre-registered exclusions	30	30	30	30

Mean age (SD)	20.33 (2.41)	27.00 (4.90)	26.63 (4.22)	27.03 (4.58)

Gender: Male/Female/Prefer not to say	6/23/1	6/24/0	14/15/1	6/25/0

Mean number of years lived outside of home country (SD)	0.00 (0.00)	1.73 (0.81)	0.00 (0.00)	0.97 (1.00)


Experiment 2 further investigated whether prior knowledge in long-term memory (LTM) influences feature binding in WM differently between the two cultural groups. The prior knowledge of the presented information was manipulated by varying the consistency between the presented shapes and colours (e.g., red apple vs. blue apple). If prior knowledge promotes WM performance by specifically facilitating extrinsic binding (Loaiza & Srokova, 2020), then prior knowledge should improve extrinsic binding more strongly than intrinsic binding. Furthermore, if there is a cultural difference in the automaticity of extrinsic binding, then this facilitation effect should be greater in Western than Eastern participants. However, if extrinsic and intrinsic binding are similarly automatic, then both cultural groups should show a prior knowledge benefit to both extrinsic and intrinsic binding memory.

## Method

### Participants

For each experiment, 60 unique Chinese and British participants (30 of each group) aged 18–35 with normal colour vision were recruited from the University of Essex or Prolific to participate. Eastern and Western participants were respectively qualified as such if they self-identified as either Chinese or British, born in China or the UK, and have not lived outside of their home countries for more than 3 years. The sample size was determined in a simulation-based power analysis (see the OSF for more details). Table 1 shows the sample characteristics and pre-registered exclusions.

Participants were compensated with partial course credit or £8/hour of participation. All participants in this experiment provided informed consent and were fully debriefed at the conclusion of the experiment. The University of Essex Ethics Subcommittee 3 approved this project (protocol number: ETH2021-0507).

### Materials and Procedure

Both experiments were programmed in PsychoPy (Peirce et al., 2019) and took place online with two phases. In the first phase, participants were invited to complete a brief demographics questionnaire and colour blindness test. Those who met the aforementioned inclusion criteria were invited to the second phase, wherein participants completed eight practice trials preceding the first block and 12 blocks of a visual WM task, with 24 critical trials per block. Participants received ongoing feedback on their performance and were offered a break after each block.

The trial sequence of Experiment 1 is presented in Figure 1A. Each trial began with a fixation cross appearing on the screen for 1s. Four different to-be-remembered shapes, either presented in four different colours on a white background (intrinsic) or white shapes on differently coloured backgrounds (extrinsic), then appeared in an invisible 2 × 2 quadrant array for 1s. After a retention interval of 1s, three test probes appeared in a single row at the centre of the screen: the target (i.e., exactly the same coloured shape as one of the originally presented items), a lure (i.e., a recombination of a presented colour and shape from the trial), and a new item (i.e., a colour or shape that were new to the trial). These probes either comprised different probe shapes (i.e., target, lure, new shape) integrated with the same target colour or the same target shape integrated with different probe colours (i.e., target, lure, new colour) to balance the nature of the decision across the features of the stimuli. Participants selected an option with their mouse at their own pace, after which a new trial began following an inter-trial interval of 1s.

The shapes of the memoranda were abstract shapes randomly drawn from a shape wheel (Li et al., 2020), with an angular separation of 72° between the four shape memoranda and a fifth shape serving as the new probe. This experiment used the same set of 12 colours, comprising two of options of the following six principal colours: red, yellow, blue, green, grey, and brown. The colours of the four memoranda presented during encoding were randomly drawn from two of these colour families (e.g., two shapes presented in yellow, and two shapes presented in blue), and the colour of the new probe was randomly selected from the remaining colour families. In this experiment, the recombined lure was a shape or colour from the other colour family in order to avoid an easy rejection of the new colour that is not in the same colour family (e.g., one yellow, blue, and grey instead of two yellows and one grey in Figure 1A). The on-screen arrangement of the memoranda during encoding and the probes during retrieval were random.

Experiment 2 was very similar to Experiment 1, with the following exceptions: First, this experiment included nameable shapes (e.g., an apple) drawn from Sutterer and Awh (2016). These images were presented in different colours (intrinsic) or on different-coloured backgrounds (extrinsic) that are consistent or inconsistent with reality (e.g., a red apple versus a blue apple; see Figure 1B). Because of the difficulty in selecting three colour shades in each colour family, we only used the “shape” probe type. In addition, the recombined lure was a shape from the same colour family to ensure that the probes were similarly consistent or inconsistent with reality depending on the condition. The new probe was also selected such that its colour was consistent or inconsistent with reality to ensure that it was a plausible option.

### Design and Analysis

Experiment 1 followed a 2 (Culture: British, Chinese) × 2 (Binding Type: Intrinsic, Extrinsic) × 2 (Probe Type: Shape, Colour) mixed design, whereas Experiment 2 followed a 2 (Culture: British, Chinese) × 2 (Binding Type: Intrinsic, Extrinsic) × 2 (Prior Knowledge: Consistent, Inconsistent) mixed design (the latter two factors of each design manipulated within-subjects). The key measure of frequency of responses of each possible category (i.e., target, lure, and new) were fitted with separate hierarchical Bayesian MPT models for each cultural group using the TreeBUGS package (Heck et al., 2018) in R. MPT models are a class of measurement models that estimate the cognitive parameters assumed to underlie the observed response frequencies. Hierarchical MPT models explicitly allow for heterogeneity across participants and trials and assume that the resulting individual parameter estimates are drawn from a population distribution. Furthermore, the Bayesian approach focuses on the posterior distribution of the parameters that are sampled using Markov-Chain Monte Carlo (MCMC) methods. The measures of interest are the mean and quantiles computed for the posterior distribution of the samples, which reflect the updated knowledge about the parameters in light of the data and given some prior beliefs. We used the default weakly informative priors of TreeBUGS that follow the recommendations of prior work (Gelman & Hill, 2006; Klauer, 2010; Matzke et al., 2015; see Heck et al., 2018 for further information).

**Figure 2:**
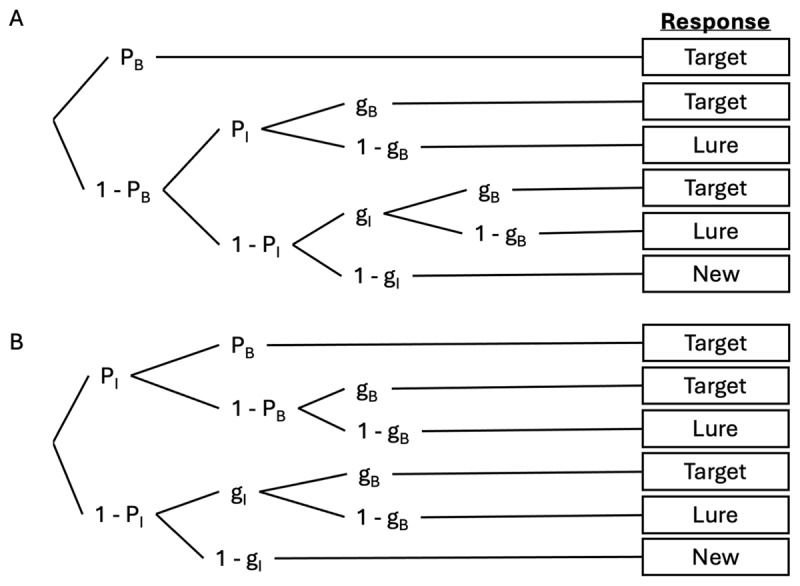
Multinomial processing tree (MPT) model, showing the independence model (Panel A) and dependence model (Panel B). The parameters shown represent the probability of binding memory (*PB*) and item memory (*PI*), and associated probabilities for guessing (*gB, gI*).

The two tested models are visually depicted in Figure 2. The difference between these models is the assumption of whether binding memory is independent of (i.e. independence model) or dependent on (i.e. dependence model) accurate memory of the individual features (i.e. item memory). The dependence model (Figure 2B) assumes that participants may correctly select the target probe when they first accurately remember the independent shape or colour features of the item (*P_I_*) as well as their correct binding (*P_B_*). In the absence of binding memory (1 – *P_B_*), then they may guess with equal probability between the target (*g_B_* = 0.5) and the lure (1 – *g_B_*). Fixing the guessing parameters (*g_B_* = 0.5 and *g_I_* = 0.5) in both models allows them to be identifiable and follows prior work (Bartsch et al., 2019; Loaiza & Srokova, 2020). Thereafter the dependence model follows the same structure as the independence model (Figure 2A) in the absence of item memory (1 – *P_I_*). Both models successfully converged (all Ȓs < 1.02) and provided good fit to the data (all *ppp*s > .05).

The comparative fit of the independence model or dependence model to the data was tested to understand the structure of these processes by comparing the deviance information criterion (DIC) of the resulting models. For the winning model, the binding memory and item memory parameter estimates were compared between cultural groups as a function of binding type (extrinsic and intrinsic) and probe type (shape and colour) in order to address the hypotheses. Specifically, given the Bayesian approach and following prior work (e.g., Bartsch et al., 2019; Loaiza & Srokova, 2020), we drew inferences by inspecting the 95% credibility interval (CI) of the mean cultural and binding difference for each of the posterior estimates of binding and item memory. The cultural difference reflects the mean difference between the two cultural groups for each type of binding and probe (i.e., extrinsic-shape, extrinsic-colour, intrinsic-shape, intrinsic-colour) or consistency (i.e., extrinsic-consistent, extrinsic-inconsistent, intrinsic-consistent, intrinsic-inconsistent). The binding difference reflects the mean difference between the extrinsic and intrinsic binding conditions for each cultural group and probe/consistency type. A 95% CI that does not contain 0 would suggest a difference between conditions for the corresponding parameter estimates.

## Results

**Figure 3:**
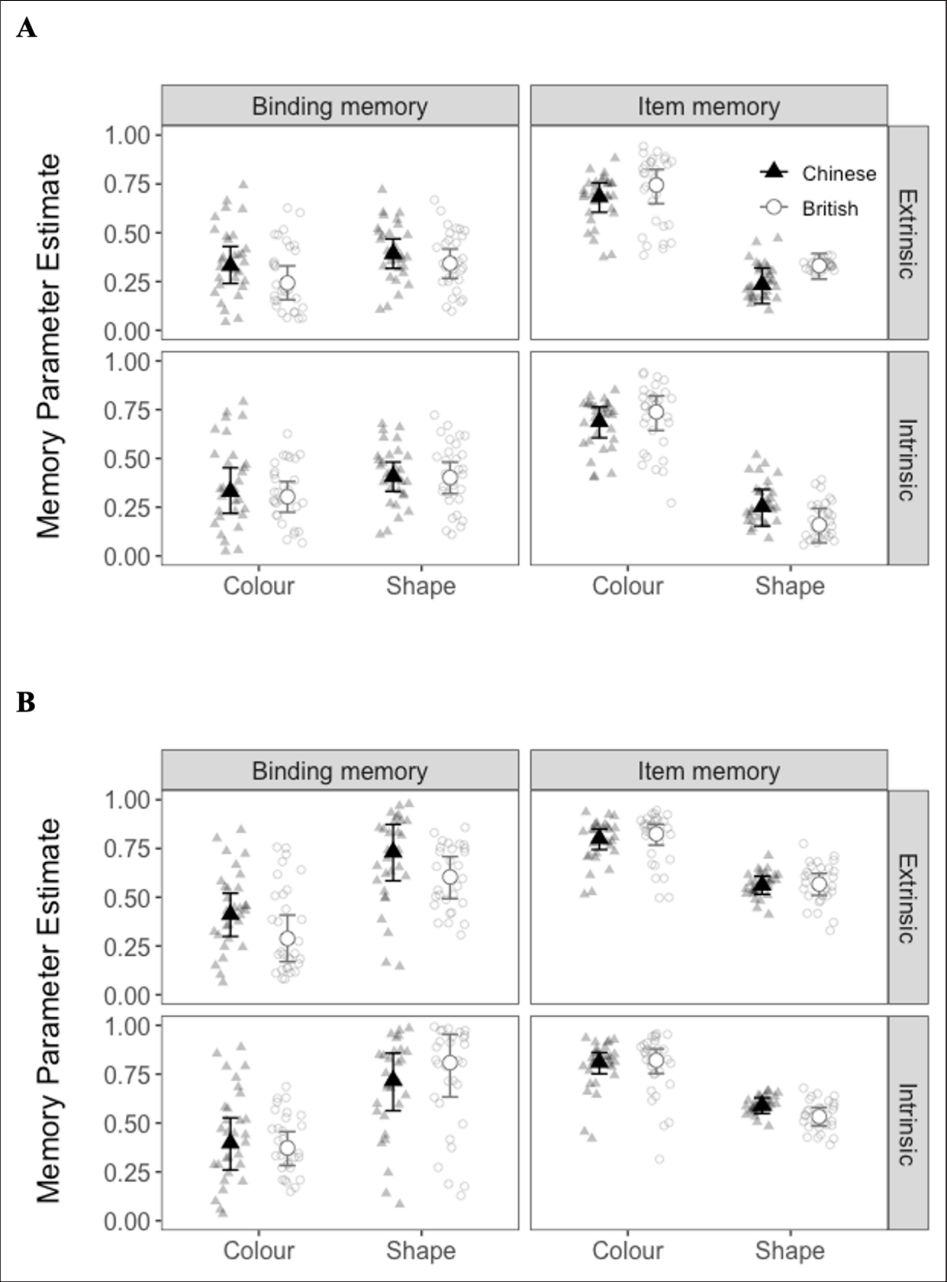
Memory Parameter Estimates for the **(A)** Independence Model and **(B)** Dependence Model for Each Cultural Group and Binding Condition in Experiment 1.

**Figure 4:**
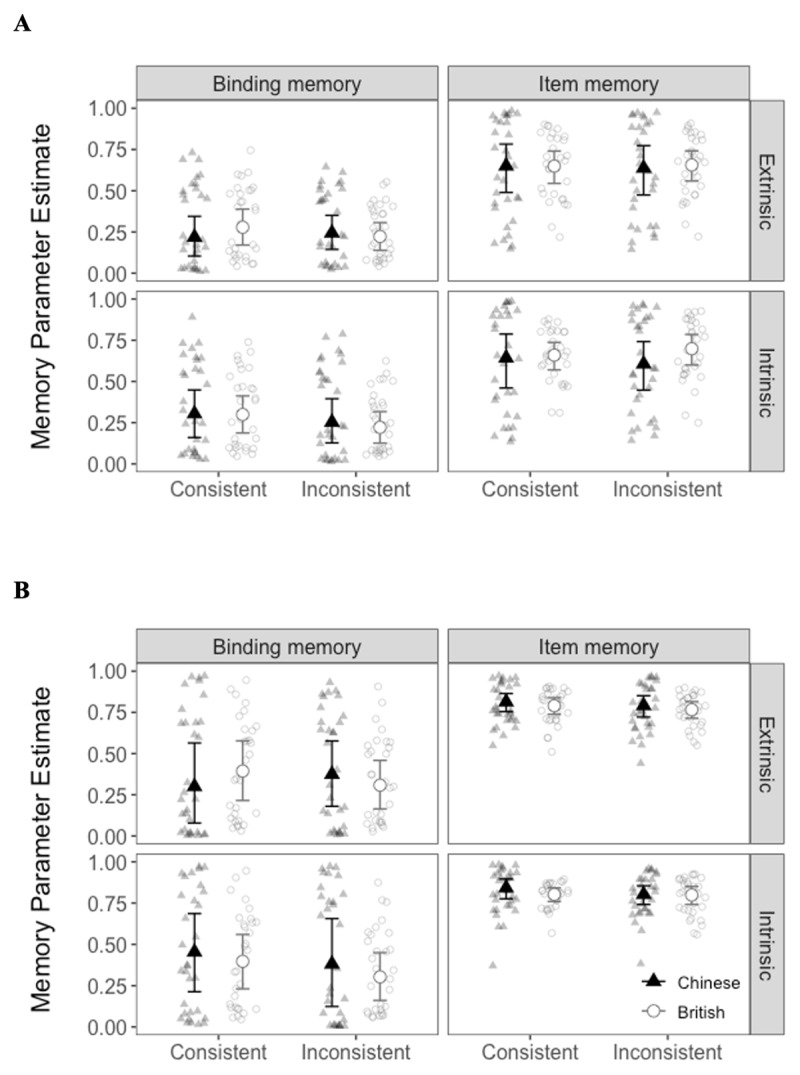
Memory Parameter Estimates for the **(A)** Independence Model and **(B)** Dependence Model for Each Cultural Group and Binding Condition in Experiment 2.

Comparison between the independence and dependence models is shown in Table 2. In Experiment 1, the dependence model fit the data of Chinese participants better than the independence model, whereas the independence model fit the data of British participants better than the dependence model. In Experiment 2, the dependence model fit the data of both British and Chinese participants better than the independence model. The results of both models were similar and are shown for comprehensiveness (see Tables 3 and 4 and Figures 3 and 4).

**Table 2:** Penalised Deviance of the Dependence and Independence Models for Both Cultural Groups in Experiments 1 and 2.


	MODEL	BRITISH	CHINESE

Experiment 1	Dependence	1305	1306

	Independence	1303	1311

Experiment 2	Dependence	1283	1316

	Independence	1289	1319


**Table 3:** Mean Posterior Estimates and Differences for the Binding Memory Parameter (Pb) [and 95% Bayesian Credibility Intervals (CI)] as a Function of Cultural Group and Binding Condition in Experiment 1.


			CULTURAL GROUP					

			BRITISH		CHINESE		*CULTURAL DIFFERENCE*	
		
	BINDING	CONDITION	MEAN	95% CI	MEAN	95% CI	MEAN	95% CI

Dependence model	Extrinsic	Colour	0.287	[0.166, 0.407]	0.412	[0.303, 0.518]	0.073	[–0.239, 0.498]

		Shape	0.605	[0.490, 0.711]	0.731	[0.590, 0.870]	–0.006	[–0.457, 0.351]

	Intrinsic	Colour	0.373	[0.282, 0.457]	0.398	[0.263, 0.527]	–0.074	[–0.304, 0.177]

		Shape	0.809	[0.631, 0.954]	0.718	[0.570, 0.855]	–0.334	[–0.726, 0.078]

	*Binding difference*	Colour	–0.084	[–0.200, 0.029]	0.064	[–0.296, 0.499]		

		Shape	–0.203	[–0.358, –0.038]	0.125	[–0.387, 0.603]		

Independence model	Extrinsic	Colour	0.243	[0.157, 0.330]	0.332	[0.240, 0.430]	0.064	[–0.143, 0.286]

		Shape	0.343	[0.266, 0.417]	0.393	[0.317, 0.469]	0.020	[–0.174, 0.230]

	Intrinsic	Colour	0.304	[0.224, 0.381]	0.331	[0.218, 0.452]	–0.033	[–0.275, 0.305]

		Shape	0.402	[0.319, 0.481]	0.406	[0.331, 0.481]	–0.040	[–0.276, 0.223]

	*Binding difference*	Colour	–0.032	[–0.165, 0.099]	0.064	[–0.279, 0.335]		

		Shape	–0.077	[–0.181, 0.027]	–0.017	[–0.288, 0.237]		


**Table 4:** Mean Posterior Estimates and Differences for the Binding Memory Parameter (Pb) [and 95% Bayesian Credibility Intervals (CI)] as a Function of Cultural Group, Binding Condition, and Prior Knowledge in Experiment 2.


			CULTURAL GROUP					

			BRITISH		CHINESE		*CULTURAL DIFFERENCE*	
		
	BINDING	CONDITION	MEAN	95% CI	MEAN	95% CI	MEAN	95% CI

Dependence model	Extrinsic	Consistent	0.392	[0.218, 0.570]	0.292	[0.079, 0.562]	0.015	[–0.421, 0.564]

		Inconsistent	0.305	[0.164, 0.449]	0.369	[0.178, 0.585]	0.104	[–0.315, 0.638]

	*Prior Knowledge Benefit*		0.086	[–0.055, 0.225]	–0.003	[–0.687, 0.689]		

	Intrinsic	Consistent	0.394	[0.238, 0.551]	0.445	[0.214, 0.684]	0.106	[–0.388, 0.605]

		Inconsistent	0.300	[0.162, 0.440]	0.371	[0.121, 0.662]	0.168	[–0.303, 0.688]

	*Prior Knowledge Benefit*		0.092	[–0.043, 0.226]	0.030	[–0.707, 0.738]		

	*Binding difference*	Consistent	–0.003	[–0.139, 0.133]	–0.094	[–0.759, 0.630]		

		Inconsistent	0.003	[–0.131, 0.139]	–0.061	[–0.754, 0.659]		

Independence model	Extrinsic	Consistent	0.279	[0.170, 0.389]	0.219	[0.104, 0.345]	0.094	[–0.291, 0.632]

		Inconsistent	0.223	[0.139, 0.307]	0.244	[0.144, 0.351]	0.157	[–0.222, 0.689]

	*Prior Knowledge Benefit*		0.058	[–0.033, 0.146]	–0.004	[–0.657, 0.653]		

	Intrinsic	Consistent	0.299	[0.187, 0.412]	0.304	[0.160, 0.449]	0.089	[–0.303, 0.605]

		Inconsistent	0.221	[0.126, 0.317]	0.253	[0.127, 0.395]	0.145	[–0.228, 0.694]

	*Prior Knowledge Benefit*		0.079	[–0.012, 0.169]	0.023	[–0.651, 0.662]		

	*Binding difference*	Consistent	–0.020	[–0.110, 0.069]	–0.015	[–0.655, 0.649]		

		Inconsistent	0.000	[–0.084, 0.088]	0.012	[–0.670, 0.692]		


For all binding-probe combinations in both Experiments, the 95% CI of the estimated mean cultural difference in all the parameters contained 0 (see the column labelled cultural difference in Tables 3 and 4). This suggests that the parameter estimates of binding and item memory were similar between the Western and Eastern participants. There was also no credible difference between extrinsic and intrinsic binding, for either colour or shape conditions in Experiment 1, and in either cultural group (see the rows labelled binding difference in Table 3 and Figure 3). This suggests that extrinsic and intrinsic binding were similar regardless of the type of probe or cultural group. Furthermore, in Experiment 2, there were no credible prior knowledge benefits for either intrinsic or extrinsic binding in either cultural group (i.e., the rows labelled prior knowledge benefit in Table 4 and Figure 4).

## Discussion

The results of Experiments 1 and 2 yielded four main findings. First, the binding memory parameter estimates were similar between intrinsic and extrinsic trials, suggesting little distinction between intrinsic and extrinsic binding. Second, there were no credible differences between the two cultural groups in extrinsic binding memory. This result goes against the prediction that extrinsic binding memory should be greater in Eastern participants based on the Analytic and Holistic framework that Easterners equally focus on foreground objects and their backgrounds given their more holistic style of thinking (Masuda & Nisbett, 2001). Third, Experiment 2 showed that there were no differences between consistent and inconsistent conditions for either cultural group, thus suggesting that prior knowledge in long-term memory does not facilitate binding memory. Finally, the results cannot distinguish whether the dependence or independence model best captures the underlying process of feature binding in WM as the penalised deviance in both models was similar in both experiments. Overall, the findings of this report show evidence against the notion that the automaticity of feature binding depends on unitisation, and that cultural differences and prior knowledge can influence feature binding.

## Data Accessibility Statement

All the experiments were pre-registered. The pre-registration, raw data, analysis scripts, and study materials are available on the Open Science Framework (OSF): https://osf.io/md2rz/.

## Footnotes

For the sake of brevity but to preserve transparency, a third incomplete experiment showing chance performance in most of the Chinese participants is reported on the OSF.
